# A Text-Book of Histology

**Published:** 1901-11

**Authors:** 


A Text-Book of Histology. Including Microscopic Technic. By A. A. Böhm, M. D., and M. Von Davidoff, M. D., of the Anatomical Institute of Munich. Edited with extensive additions to both Text and Illustrations, by G. Carl Huber, M. D., Junior Professor of Anatomy and Director of the Histological Laboratory University of Michigan. Authorized Translation from the Second Revised German Edition. By Herbert H. Cushing, M. D., Demonstrator of Histology and Embryology, Jefferson Medical College, Philadelphia. With 351 illustration. Pp. 500. Price, $3.50. Philadelphia: W. B. Saunders & Co.; London: 161 Strand, W. C. 1900.

This is the first American edition from the second German edition, and represents histology as taught at Munich, the center of the world on that subject. The American edition has retained all of the good parts of the second German edition, and added much that has come from the American laboratories, especially as relates to the motor and sensory nerve endings, and on the spinal and sympathetic ganglia. Expansion of the text on the innervation of glands and organs, and especially as applied to the function of tissues, is a most important and interesting part of the book. Over one hundred appropriate illustrations have also been added. There is no question as to the high character of the work. The American student will no doubt take advantage of this edition. T. J. B.

